# Is presence of children or youth a farm workplace injury risk factor on Irish farms?

**DOI:** 10.3389/fpubh.2022.1074673

**Published:** 2023-01-18

**Authors:** John McNamara, Mohammad Mohammadrezaei, Emma Dillon, David Meredith

**Affiliations:** ^1^Teagasc—Irish Agriculture and Food Development Authority, Farm Health and Safety Knowledge Transfer Unit, Kilkenny, Ireland; ^2^Teagasc—Irish Agriculture and Food Development Authority, Rural Economy Development Programme, Dublin, Ireland; ^3^Teagasc—Irish Agriculture and Food Development Authority, Rural Economy Development Programme, Galway, Ireland

**Keywords:** children and youth, dairying, farm survey, Ireland, risk factor, workload

## 1. Introduction

This paper describes identifying a farm workplace injury risk factor associated with the presence of children/youth on Irish farms. Under Irish safety, health and welfare at work legislation a person in the age category of 0–18 years old or still at school are considered a child or young person. However, as the UN defines youth as persons between the ages of 15 and 24 years, this definition related to youth age is used in this paper (1). The presence of children/youth has been overlooked in past studies seeking to identify farm workplace injury risk factors in agriculture. A risk factor has been described as a factor related to the probability of an injury, which allows a population to be subdivided into risk category groups based on the presence or absence of the identified risk factor (2). Gaining information to allow risk factor(s) identification is a prerequisite to developing effective and tailored prevention strategies (2).

To identify a risk factor, securing data related to farm workplace injury occurrence and associated population factors is necessary. A means of gaining data on farm workplace injuries in Ireland has been by use of the National Farm Survey (NFS) operated by Teagasc—the Irish Agriculture and Food Development Authority. The NFS is part of the European Union (EU) Farm Accounts Data Network (FADN), which collects physical, technical and financial data on a nationally representative sample of farms throughout Europe. In addition to core FADN data, the NFS collects data pertaining to sustainability, including social aspects such as health and safety. As such, the NFS has previously been used to gain estimates of farm workplace injury levels through an additional survey mechanism (3). Farm workplace injury survey data gained has been successfully analyzed in association with core NFS data, which provides a considerable range of socio-economic variables, to explore farm workplace injury risks.

Previous research indicates that both behavioral and farm infrastructural factors are likely to be associated with farm workplace injury levels (3, 4). In particular, issues such as working long hours, rushing and tiredness along with under investment in safety related infrastructure and mechanization are likely to be associated with farm workplace injury occurrence. In addition, there is some evidence that indirectly highlights the presence of children/youth in particular in summer, as the busiest time on farms, when increased workload might be a factor associated with farm workplace injury occurrence (5). In Ireland, an average dairy farm enterprise is recognized as being relatively more profitable (6) and labor intensive (7) than other enterprises, while farm operators work long hours under multiple stressors (8). A previous study based on NFS data indicated that among enterprises dairy farms had the highest level of farm workplace injuries (9). Dairying attracts a younger and farm development-oriented farming population (10) and accordingly, farm families with a dairying enterprise are more likely to have children/youth in the household. While previous NFS based research in the 1990's in Ireland (11) pointed to the possibility of “having children/youth” as a farm workplace injury risk factor, the “presence of children/youth” has been overlooked in almost all previous studies. In contrast, work-related and economic risk factors including gender, age, family size and farm enterprise have been considered in farm workplace identification (12, 13). Therefore, to the best of our knowledge, to date, “presence of children/youth” in all farm enterprises and on dairy farms in particular, as a potential farm workplace injury risk factor has not been well studied.

Accordingly, this study aims to examine the following two main hypotheses:

H1: having children/youth aged under 24 years (H1a) in general, and aged <5 (H1b), 5–15 (H1c), 16–19 (H1d), 20–24 (H1e) in particular within this age range represent a farm workplace injury on Irish farms.
H2: having households with/without member(s) aged 1–24 (H2a) in general, and <5 (H2b), 5–15 (H2c), 16–19 (H2d), 20–24 (H2e) in particular is associated with a higher farm workplace injury occurrence on dairy farms.

## 2. Methods

### 2.1. Data collection and measures

Data relating to farm workplace injury was collected through the NFS in 2017. Teagasc, as a statutory body is permitted to conduct such a survey without ethics approval. However, the survey complies with “conditions for consent” under the EU General Data Protection (14).

For comparative purposes, the survey was designed to match those previously conducted through the NFS related to farm workplace injuries (3). A farm workplace injury occurrence was defined as “a farm work related event (including a farm work related road traffic injury) which led to physical harm causing bodily injury” in the previous 5-year period. In total, NFS recorders completed 893 injuries survey questionnaires through face-to-face interviews with individual Farm Operators with main responsibility for the operation of the farm. In this study, to examine the study hypotheses, data related to age profile of farm household members (ordinal scale) and farm enterprise (nominal scale) was combined with farm workplace injury occurrence (binary variable) for correlation analysis. Data for all farms (100%) that participated in the NFS in 2017 is included in this survey.

### 2.2. Data analysis

Descriptive analysis using frequencies and cross-tabulation analysis were applied to test the study hypotheses. To this end, examining H1a, the correlations between the presence of children/youth (aged 1–24) (Table 1) in farm households associated with occurrence of a farm workplace injury were estimated using cross-tabulation analysis. Equally, Chi-square analysis was then applied to demonstrate the association between having children/youth (aged 1–24) (H1a), children aged <5 (H1b), 5–15 (H1c), 16–19 (H1d), 21–24(H1e) and FWI occurrence reported in the past 5 years (binary dependent variable (yes /no). The same approach was applied for examining H2a and H2b. To estimate the predictive power of dependent variables on the injury occurrence, the crude odds ratio was estimated (15). The data set was analyzed using the statistical package SPSS version 21 for windows.

**Table 1:** Farm workplace injury occurrence associations with farming enterprise and presence/absence of children/youth by age range in farm households.

**Variables (nominal)**	**Farm injury occurrence** **(*****N*** = **893)**				**Statistical analysis**		

	**Yes** **(113, 12.6%)**		**No** **(780, 87.4%)**				
**Farm enterprise (** ***n*** **, %)**	***n***	**%**	***n***	**%**	**Crude odds of injury**	**Test**	***P*** **-value**
Dairy farming (313, 35.1%)	54	16.9	259	83.1	0.21	***10.84***	***0.05***
Cattle other (217, 24.3%)	24	11.1	193	88.9	0.12		
Cattle rearing (151, 16.9%)	10	6.6	141	93.4	0.07		
Sheep farming (126, 14.1%)	16	12.7	110	87.3	0.05		
Crop production (72, 8.1%)^*^	<10	11.1		89.9	0.13		
Others (14, 1.6%)^*^	<10	14.3		85.7	0.08		
**Having children (aged**<**5)**						0.45	0.5
Yes (51, 5.7%)^*^	< 10	15.7		84.3	0.19		
No (842, 94.3%)	105	12.5	737	87.5	0.14		
**Having children (aged 5–15)**							
Yes (162, 18.1%)	34	21.0	128	79.0	0.43	***12.435***	***0.001***
No (731, 81.9%)	79	10.8	652	89.2	0.12		
**Having children/youth (aged 16–19)**						0.237	0.60
Yes (143, 16%)	20	14	123	86	0.16		
No (750, 84%)	93	12.4	657	87.6	0.14		
**Having children/youth (aged 20–24)**							
Yes (132, 14.8%)	22	16.7	110	83.3	0.2	2.25	0.11
No (761, 85.2%)	91	12	670	88	0.14		
**Having children/youth (aged 1–24)**							
Yes (353, 39.6%)	61	17.3	292	82.7	0.21	***11.305***	***0.001***
No (540, 60.4%)	52	9.6	488	90.4	0.10		

* Observations less than 10 cannot be displayed due to data confidentiality.

## 3. Results

Almost forty percent (39.6%) of family farms indicated having *children/youth (aged 1–24)* in their family households. However, considerable variation existed in the level of children/youth in farm households with dairy farms having a much *higher proportion of having children/youth* compared to other enterprises (16). The two age cohorts including 5–15 (24.5%) and 20–24 (20.7%) formed the main proportion of the children/youth population on dairy family farms.

A “farm workplace injury occurrence” during the previous 5 years was reported by 113 family farms (12.6%) (Table 1), where nearly half indicated occurrence on dairy farms (*n* = 54, 46.9%). The study data identifies dairy farms as the most dangerous farm enterprise regarding farm workplace injury occurrence and it also has the highest proportion of children/youth (47.9%) where 24.6% of farms had children aged 5–15 in their households. Chi-square analysis showed that dairy farmers reported farm workplace injury occurrence as nearly twice the level of other enterprises (Chi-square = 10.84, *P* = 0.05) (Table 1). Regarding the association between having children/youth and farm workplace injury occurrence, there was no significant difference between farm families who had children/youth aged 5<, 16–19, and 20–24 (Table 1). Therefore, H1b, H1c, and H1e are rejected. Interestingly, we found that family farms with children/youth aged 1–24 (39.6%) in their household reported twice as many “farm workplace injury occurrences” compared to families without youth/children (Chi-square = 11.305, *P* = 0.001). as such, H1a is approved. Furthermore, confirming H1b, the study data shows that “farm workplace injury occurrence” was four times higher for family farms that had children aged 5–15 in their household compared to other family farms (Chi-square = 12.435, *P* = 0.001). Thus, having children/youth, in general, and children aged 5–15 in particular on a family farm is a major risk factor associated with farm workplace injury occurrence. Thus, the study data indicate that having children/youth and having children in the 5–15 age category, in particular should be considered as an important farm workplace injury risk factor, particularly on dairy farms.

## 4. Discussion/Conclusions

The study findings indicate that two elements of H1 be accepted, namely, the presence of having children/youth (1–24 years old) (H1a) in the farm household and 5–15 year olds (H1c), in particular, is associated a farm workplace injury risk. This novel finding has not been reported previously, to our knowledge. The possible reason why this risk may happen on farms with children/youth could be due to the presence of younger parents (10) who are involved in farm development and farming that is more intensive or off-farm employment. Thus, the risk may be based on the parental stage in life and farm development activities and/or workload. International studies have indicated that increased farm work time increases farm workplace injury rate (17–20) and also in Irish studies (21, 22). In particular, one previous Irish study using NFS data has indicated generally that farm scale, workload, and under investment, influence farm operator farm workplace injury rate (3). This study (3) identified farms where both farm operator and spouse engaged in off-farm employment as a risk factor for farm operator injury risk. A further possible explanation is that childcare, particularly in the 5–15-age category, may require time, which accordingly causes increased work time demands on adult family members. A previous Irish study has indicated that farmers believe that increased “work time” demand is a major contributory factor for farm injury occurrence (4).

The study data indicates that both elements of H2 should be accepted. Namely, the presence of children/youth in farm households (H2a) and 5–15 year olds (H2c), on dairy farms in particular, is associated a farm workplace injury risk. This is unsurprising as this enterprise is associated with high workload, long working hours and associated stressors and having children could cause higher non-task based work hours spent by parents, which thus increases the risk of FWIs.

Identification of a risk factor indicates that a particular segment of a population has a heightened risk. Accordingly, study of factors leading to the heightened risk is potentially valuable to identify reasons for the increased risk and to identify control measures. The study of farm workplace injury associated with children/youth in farm households has received limited examination in the scientific literature. Given this knowledge gap and the novelty of the findings of this study, further research is warranted on the association between farm workplace injury risk factors and the presence of children/youth in farm families. One way to conduct such research could be a further examination of NFS core data to investigate farm and socio-economic variables related to this issue. A further approach could be to conduct qualitative research using such approaches as interviews, focus groups or case studies among a sample of adult farm family members with children/youth who are in the identified high risk sub population. This approach would gain a nuanced account of the safety and health challenges associated with both working and child-rearing in tandem on Irish farms. Overall this paper in identifying a risk factor indicates new approaches for both research and outreach to reduce the level of farm workplace injuries on farms where children/youth are present.

## Author contributions

JM identified the paper concept, sourced the study data from NFS, and prepared the initial manuscript draft. MM conducted the statistical analysis of data in the paper and contributed to writing the manuscript draft. DM and ED reviewed the draft manuscript and provided material for inclusion in the paper. All author read the penultimate version of the paper and provided edits and are in agreement with its content.
